# CD4^+^CCR8^+^ Tregs in ovarian cancer: a potential effector Tregs for immune regulation

**DOI:** 10.1186/s12967-023-04686-3

**Published:** 2023-11-10

**Authors:** Shuna Liu, Ziqi Tao, Jianfang Lou, Rong Li, Xin Fu, Juan Xu, Ting Wang, Lei Zhang, Wenwen Shang, Yepeng Mao, Fang Wang

**Affiliations:** 1 Department of Laboratory Medicine, The First Affiliated Hospital with Nanjing Medical University, No. 300 of Guangzhou Road, Nanjing, 210029 China; 2 Branch of National Clinical Research Center for Laboratory Medicine, Nanjing, 210029 China; 3 Department of Gynecology, Women’s Hospital of Nanjing Medical University, Nanjing, 210004 China; 4 Department of Laboratory Medicine, The Affiliated Taizhou People’s Hospital of Nanjing Medical University, Taizhou, 225300 China; 5 Department of Gynecology, The Affiliated Huaian NO.1 People’s Hospital of Nanjing Medical University, Huaian, 223300 China

**Keywords:** Ovarian cancer, Regulatory T cells, CCR8, Gene expression profile, Chemokine

## Abstract

**Background:**

Tregs are key drivers of immunosuppression in solid tumors. As an important chemokine receptor on Tregs, the regulatory effect of CCR8 on tumor immunity has received more and more attention. However, the current research on CCR8 in the immune microenvironment of ovarian cancer has not been clear.

**Methods:**

Bioinformatics analysis was used to compare the transcriptome differences between CD4^+^ T cells in the peripheral circulation and infiltrated in ovarian tumor tissues. RT-PCR was used to detect the expression levels of chemokine receptor-related differential genes on CD4^+^ T cells in peripheral blood and ovarian tumor tissues. Multiparameter flow cytometry was used to detect the proportion and phenotypic characteristics of CD4^+^CCR8^+^ Tregs and CD4^+^CCR8^−^ Tregs in different sample types. The expression level of CCR8 ligands was detected at multiple levels. To explore the important role of CCR8-CCL1 and CCR8-CCL18 axis in the migration and invasion of CD4^+^CCR8^+^ Tregs into ovarian tumor tissues by establishing a chemotaxis system in vitro.

**Results:**

In this study, significantly different gene expression profiles were found between peripheral circulating CD4^+^ T cells and infiltrating CD4^+^ T cells in ovarian tumor tissues, in which chemokine-chemokine receptor signaling pathway was significantly enriched in all three groups of differential genes. The expression level of CCR8 in infiltrating CD4^+^ T cells of ovarian cancer tissue was significantly higher than that in peripheral blood of healthy controls and ovarian cancer patients, and high expression of CCR8 was significantly correlated with advanced tumor stage and poor differentiation. CD4^+^CCR8^+^ Tregs are the main type of infiltrating CD4^+^ Tregs in ovarian tumor tissues, which have stronger immunosuppressive phenotypes, secrete more inhibitory cytokines and have stronger proliferation ability. The ligands CCL1 and CCL18 corresponding to CCR8 were significantly overexpressed in ovarian tumor tissues, and the CCR8-CCL1 and CCR8-CCL18 axis played a key role in the migration and infiltration of CD4^+^CCR8^+^ Tregs into ovarian tumor tissues.

**Conclusions:**

The results of this study may help to understand the phenotypic characteristics and recruitment process of Tregs in the tumor, and provide new ideas for improving the immunosuppressive status of the ovarian cancer microenvironment.

**Supplementary Information:**

The online version contains supplementary material available at 10.1186/s12967-023-04686-3.

## Introduction

Ovarian cancer (OC) is the type of malignancies of reproductive system with the highest mortality among female, and its incidence is second only to cervical cancer and uterine body cancer [1]. Due to its insidious onset, rapid progression and lack of effective early screening methods, most patients have been diagnosed with advanced stage [2]. In recent years, immunotherapy has become a new type of tumor treatment that has attracted much attention. A large number of studies have developed a variety of therapeutic strategies for all aspects of tumor immunity, but the therapeutic effects are still not very satisfactory [3–5].

Immunosuppression of the tumor microenvironment (TME) is a key obstacle to tumor immunotherapy [6]. Tregs are important members of immunosuppressive cells that exert immunosuppressive function through mechanisms independent of cell contact (secretion of inhibitory cytokines and metabolic disruption) or dependent on cell contact (regulation of antigen-presenting cell function and mediating the dissolution or apoptosis of target cells) and which is major obstacle to driving effective anti-tumor specific immune responses [7–14].

Chemokine-chemokine receptor signaling mediated recruitment is the main mechanism of Tregs infiltration in tumors [15, 16]. Chemokines are structurally similar to cytokines in that they regulate cellular signaling and transport by interacting with a subset of seven transmembrane G-protein-coupled receptors called chemokine receptors [17–19]. Studies have shown that Tregs express a variety of chemokine receptors, such as CCR4, CCR5, CCR8 and CCR10, which can respond to a variety of chemokines released during tumor growth, and then participate in the migration of Tregs to tumor tissues [20–22]. Blocking the interaction between chemokines and chemokine receptors could reduce the infiltration of Tregs into tumor sites, thereby reactivating the suppressed anti-tumor immune response.

In this study, we compared differences in gene expression profiles between peripheral circulation and tumor tissue infiltrated CD4^+^ T cells, and analyzed the correlation between the level of CCR8 expression on CD4^+^ T cells from different sample sources and clinical characteristics. The proportion and phenotypic characteristics of CD4^+^CCR8^+^ Tregs in peripheral blood of healthy individuals, peripheral blood of OC patients and OC tissues were clarified as well as the effect of ovarian cancer microenvironment on CD4^+^CCR8^+^ Tregs recruitment.

## Materials and methods

### Patients and specimens

Blood and tissue specimens were collected from 39 OC patients who received treatment at Nanjing Maternal and Child Health Care Hospital from July 2020 to January 2022. After surgical resection, tissue specimens were quickly divided into sections and stored in medium for transport at low temperature or fixed with 4% paraformaldehyde and being embedded in paraffin for reserve. All OC patients in the study were confirmed by histopathology and had not undergone surgery, chemoradiotherapy or other immunotherapy prior to sample collection. Peripheral blood samples of 22 healthy controls were obtained from healthy donors at the corresponding period, excluding immune-related diseases. This study was authorized by the Ethics Committee of the First Affiliated Hospital of Nanjing Medical University with the written informed consent from all patients.

### Data analysis of gene expression profile

CapitalBio Technology Human Array v4 chip hybridization results were preprocessed and analyzed by FeatureExtraction software. GeneSpring GX software was used to calculate and compare differences in gene expression differences and statistical significance *P* values. Data normalization and quality control (QC) analysis were performed on each sample. Cluster3.0 software was used for cluster analysis and graphical representation. Based on the grouping information, the difference rate was compared to obtain the differential genes. KEGG Pathway analysis was performed on differentially expressed mRNAs.

### Isolation of CD4^+^ T cells

Peripheral blood mononuclear cells (PBMCs) and tumour-infiltrating lymphocytes (TILs) were isolated by using density gradient centrifugation with Ficol-Hypaque kit (TBD, Tianjin, China) and Percoll kit (GE, Germany), respectively. CD4^+^ T cells from circulating peripheral blood lymphocytes (PBLs) and TILs were isolated by using CD4 positive isolation kit (Miltenyi Biotec, Germany). The purity of CD4^+^ T cells was confirmed by flow cytometry to be over 95%.

### RNA Isolation and real-time quantitative PCR

Total RNA was extracted from CD4^+^ PBLs and CD4^+^ TILs by using the RNeasy Micro kit (Qiagen, Dusseldorf, Germany), total RNA from tissue samples was extracted by using Trizol (Invitrogen, CA, USA). The RNA product was then reverse-transcribed into cDNA by using Prime Script RT Master Mix (Takara, Otsu, Japan). The mRNA expression levels of chemokine receptor pathway-related differential genes and CCR8-related ligands were analyzed by using SYBR Green qPCR Master Mix (Takara, Otsu, Japan) and ABI 7500 real-time PCR (Life Technologies, Foster City, CA, USA). The primer sequence was shown in Table 1.

**Table 1:** List of PCR primer sequences

Gene name	Forward primer(5′ → 3′)	Reverse primer(5′ → 3′)
CCR1	CAGCCTTCACTTTCCTCACG	AACGGACAGCTTTGGATTTCTT
CCR2	CCACATCTCGTTCTCGGTTTATC	CAGGGAGCACCGTAATCATAATC
CCR3	TCCTTCTCTCTTCCTATCAATC	GGCAATTTTCTGCATCTG
CCR5	CTTCTGGGCTCCCTACAACA	CAGATATTTCCTGCTCCCCA
CCR6	TTCAGCGATGTTTTCGACTCC	GCAATCGGTACAAATAGCCTGG
CCR7	TGAGGTCACGGACGATTACAT	GTAGGCCCACGAAACAAATGAT
CCR8	CAAGCCCCTGTGATGCGGAAC	AGACCACAAGGACCAGGATGACC
CCR9	ATGTCAGGCAGTTTGCGAG	TGCAGTACCAGTAGACAAGGAT
CCR10	TGCTGGATACTGCCGATCTACTG	TCTAGATTCGCAGCCCTAGTTGTC
CXCR3	CCGTCCAGTGGGTCTTTGG	AGGGCTCCTGCGTAGAAGTTG
CXCR4	TGTCATCTACACAGTCAACCTC	CAACATAGACCACCTTTTCAGC
CXCR5	TACCCGCTAACGCTGGAAATGGAC	CACGGCAAAGGGCAAGATGAAGAC
CXCR6	ATGCCATGACCAGCTTTCACT	ATGCCATGACCAGCTTTCACTG
CCL1	AATACCAGCTCCATCTGCTCCAA	GAACCCATCCAACTGTGTCCAAG
CCL8	TGGAGAGCTACACAAGAATCACC	TGGTCCAGATGCTTCATGGAA
CCL16	GCCCACTGAGAGGATGAAGG	TACTTCAGGCAGCAGTTGGG
CCL18	TGCCCAGCATCATGAAGG	TCAGGCATTCAGCTTCAGG
β-actin	GAGCTACGAGCTGCCTGACG	GTAGTTTCGTGGATGCCACAG

### Flow cytometry

Surface staining of isolated PBMCs and TILs were performed using fluorescein conjugated antibodies according to the manufacturer's instructions. The antibodies used included Anti-CD3-Percp-Cyanine, Anti-CD45-PE/Cyanine7, Anti-CD4-FITC, Anti-CD39-APC, Anti-PD-1-APC, Anti-CTLA4-APC (all from Biolegend, CA, USA), Anti-CCR8-BV421 (BD Biosciences, CA, USA). After being cleaned, fixed and penetrated, Anti-IL-10-APC, Anti-TGF-β-APC and Anti-Ki-67-APC (all from Biolegend, CA, USA) and Anti-Foxp3-PE (BD Biosciences, CA, USA) were used to label intracellular cytokines, Ki-67 and Foxp3.

### Western blot

Whole cell lysates of tissue samples were prepared, and then CCL1 Rabbit polyclonal Antibody, CCL18 Rabbit polyclonal Antibody (Abcam, Cambrige, UK), GAPDH Rabbit Monoclonal Antibody and Horseradish peroxidase labeled Goat Anti-Rabbit IgG(H + L) (Biyuntian Biotechnology, Shanghai, China) were used to label the target protein.

### Immunohistochemistry

Cancerous tissues and adjacent cancerous tissues were sectioned after formalin fixation and paraffin embedding. Antibodies used for labeling process included CCL1 Rabbit polyclonal Antibody and CCL18 Rabbit polyclonal Antibody (Abcam, Cambrige, UK). DAB kit was used for coloration and digital pathological biopsy scanner was used to scan and fetch the images. 10 counting areas were randomly selected under the low magnification microscope, and 200 tumor cells were counted under the high magnification microscope, and the number of positive cells was recorded to calculate the positive rate of cell staining. The result of histochemical staining was judged by the product of the positive rate of cell staining and the score of color depth.

### ELISA

Cell suspensions from cancerous tissues and adjacent cancerous tissues were prepared with a concentration of 1 × 10^6^ cells/mL. 4 mL cell suspension was added into the cell culture flask and cultured for 48 h in a 5%CO_2_ incubator at 37℃. The supernatant in the flask was then collected and centrifuged to remove debris for further use. I-309/CCL1 Human ELISA Kit (Invitrogen, Carlsbad, CA, USA) and Human CCL18/PARC ELISA Kit (MUKTI SCIENCES, Hangzhou, China) were used to detect the levels of CCL1 and CCL18 in different culture supernatants.

### Cell sorting and amplification

PBMCs were stained with fluorescein conjugated antibodies including Anti-CD4-FITC, Anti-CD25-APC, Anti-CD127-PE (all from Biolegend, CA, USA) and Anti-BV421-CCR8 (BD Biosciences, CA, USA), and sorted by FACS Aria II cell sorter (BD Biosciences, CA, USA) was used for sorting. CD4^+^CCR8^−^ Tregs and CD4^+^CCR8^+^ Tregs obtained by the sorter were washed and re-suspended in X-VIVO15 medium (LONGZA, Basle, Switzerland) with 5% human AB serum (Gemini, Woodland, CA, USA) and 500 U/ml IL-2 (Peprotech, Rocky Hill, NJ, USA), and then 1 × 10^5^ cells/well were added into 96-well round plates, followed by 20 μL anti-CD3/CD28 coated microbeads (Invitrogen, Carlsbad, CA, USA) added into each well.

### Chemotactic assay

CD4^+^CCR8^−^ Tregs, CD4^+^CCR8^+^ Tregs and tissue culture supernatants were collected. Recombinant human CCL1/I-309 and recombinant human CCL18/MIP-4 were used to construct mediums with gradient concentrations of chemokines (0.01 ng/mL, 0.1 ng/mL, 1 ng/mL, 10 ng/mL, 100 ng/mL). In the lower chamber of the co-culture system, tissue culture supernatants or mediums containing chemokines of different concentrations (CCL1/CCL18) were added respectively, and CD4^+^CCR8^−^ Tregs or CD4^+^CCR8^+^ Tregs were added respectively in the upper chamber. The plate was cultured in a 5%CO_2_ incubator at 37 ℃. After 150 min, the number of cells chemotactic to the lower chamber was counted and the chemotactic index of each group was calculated as well.

### Data analysis

Data were analyzed with GraphPad prism 7.0 software (GraphPad Software Inc., San Diego, CA, USA). Comparisons between different groups were calculated using Student’s t-test or the non-parametric Mann–Whitney U test. The association between variables and clinical characteristics was evaluated by Chi-square or Fisher exact test. Experiments were independently repeated at least three times. The data were expressed as means ± standard deviation (SD) and *P* values < 0.05 were considered statistically significant (Additional files 2, 3 Figures S2, S3).

## Results

### CCR8 expression on CD4^+^ TILs and CD4^+^ PBLs

**Fig. 1:**
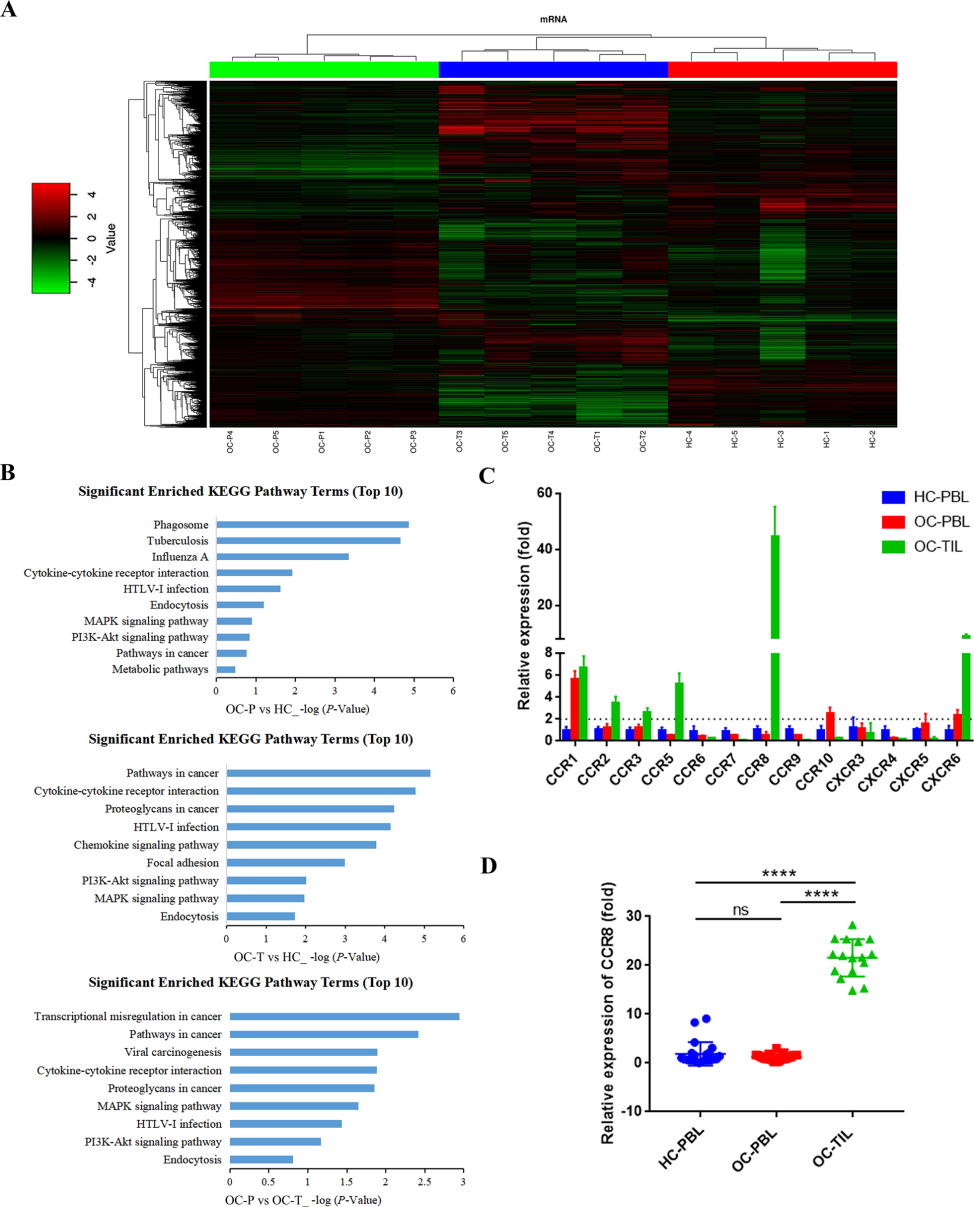
Differential expression profiles between CD4^+^ PBLs and CD4^+^ TILs **A** Clustering analysis of mRNAs that are differentially expressed in CD4^+^ PBLs from healthy controls (HC), CD4^+^ PBLs from OC patients (OC-P), and CD4^+^ TILs from OC patients (OC-T). Each column represents a sample and each row represents a specific gene. Those labeled in red are upregulated genes, labeled in green are downregulated genes and labeled in black are non-significantly different genes. The color scale on the left is a comparison table of values and colors (log scale 10, from − 4 to + 4).** B** Significant enrichment top 10 pathways within signal transduction pathways in the three groups. **C** Expression of chemokine receptor-related differential genes in CD4^+^ T cells of HC-PBL (n = 10), OC-PBL (n = 10) and OC-TIL (n = 10) groups. **D** Expression levels of CCR8 on CD4^+^ T cells of HC-PBL (n = 22), OC-PBL (n = 27) and OC-TIL (n = 16) groups. Data are presented as means ± SD and analysed with Student’s *t*-test. (HC-PBL group: CD4^+^ PBLs from healthy controls; OC-PBL group: CD4^+^ PBLs from OC patients; OC-TIL group: CD4^+^ TILs from OC patients; **** *P* < 0.0001.)

CD4^+^ T cells from peripheral blood of 5 healthy controls, peripheral blood and tumor tissues of 5 OC patients were extracted. Gene expression profiles of CD4^+^ T cells from the three sources were detected and analyzed by using microarrays. And correlation analysis of each group showed that all groups had a good intra-group correlation (Additional file 1: Figure S1B). Screening criteria for significant differential mRNAs were: FC (Fold change) value of mRNA between two groups  ≥ 2 and *P* ≤ 0.05. Clustering analysis and scatter plot analysis showed that the three groups of CD4^+^ T cells had significant differential mRNA expression profiles from each other (Fig. 1A, Additional file 1: Figure S1A). Further KEGG enrichment analysis showed that the signal transduction pathway was the most significantly enriched in all three groups (Additional file 1: Figure S1D). Next, we enriched the differential genes contained of signaling pathways in each group again to clarify the specific pathways they are involved in. As shown in Fig. 1B, the top 10 most significantly enriched pathways in each group were list, in which the chemokine-chemokine receptor signaling pathway showed significant enrichment in all three groups. As an important member of the cell signaling pathway, the chemokine-chemokine receptor signaling pathway is involved in regulating the tumor immune microenvironment by mediating the targeted migration of multiple immune cells. Since multiple specific chemokine receptors on T cells are important factors in mediating T cell migration, we selected chemokine receptor-related genes that had significantly differential expression in each group (Additional file 4: Table S1) and then verified their expression levels. As shown in Fig. 1C, the expression levels of the above genes in clinical specimens were consistent with the trend of microarray, among which the differential expression of CCR8 was the most significant (all groups n = 10), thus CCR8 was selected for further investigation. We then further analyzed the expression level of CCR8 in different clinical specimens by increasing the number of specimens in each group. As shown in Fig. 1D, the expression level of CCR8 on CD4^+^ TILs was significantly higher than that on CD4^+^ PBLs, and the differences were all statistically significant (*P* < 0.0001).

### Association of CCR8 expression on CD4^+^ TILs and CD4^+^ PBLs with clinical characteristics of OC patients

According to the median value of CCR8 expression on CD4^+^ TILs, 33 OC patients were divided into CCR8^low^ group and CCR8^high^ group and the correlation between CCR8 expression and clinical characteristics of OC patients were analyzed. As shown in Table 2, CCR8 expression on CD4^+^ TILs of stage III-IV patients were significantly higher than that of stage I-II patients, and CCR8 expression on CD4^+^ TILs of patients with poor differentiation were significantly higher than that of patients with well-moderate differentiation, and the differences were statistically significant (*P* < 0.05). Similarly, according to the median value CCR8 expression on CD4^+^ PBLs, we divided 39 OC patients into CCR8^low^ group and CCR8^high^ group. The correlation analysis between CCR8 expression and clinical characteristics showed that no significant association was observed between CCR8 expression on CD4^+^ PBLs and patients' age, tumor size, tumor type, FIGO stage, tumor differentiation grade, lymph node metastasis, distant metastasis, serum CA125 level, serum HE4 level and the presence of ascites (*P* > 0.05) (Additional file 5: Table S2).

**Table 2:** Correlations between the expression of CCR8 on CD4^+^ TILs and the clinicopathologic characteristics of OC patients

Clinical variables	CCR8^low^ (%)	CCR8^high^ (%)	*P *value
Sample size	17 (51.5)	16 (48.5)	
Age (year)			
< 50 years	6 (46.2)	7 (53.8)	0.7283
≥ 50 years	11 (55)	9 (45)	
Tumor size (cm)			
< 5 cm	0 (0)	0 (0)	> 0.9999
≥ 5 cm	17 (51.5)	16 (48.5)	
Histologic type			
Serous carcinoma	14 (58.3)	10 (41.7)	0.325
Endometrioid cancer	1 (50)	1 (50)	
Clear cell carcinom	1 (16.7)	5 (83.3)	
FIGO stage			
I–II	14 (66.7)	7 (33.3)	0.0324*
III–IV	3 (25)	9 (75)	
Differentiation			
Well to moderate	15 (68.2)	7 (31.8)	0.0104*
Poor	2 (18.2)	9 (81.8)	
Lymphatic metastasis			
No	5 (35.7)	9 (64.3)	0.1663
Yes	12 (63.2)	7 (36.8)	
Distant metastasis			
No	3 (27.3)	8 (72.7)	0.0707
Yes	14 (63.6)	8 (36.4)	
CA125 (U/mL)			
< 200 U/mL	3 (33.3)	6 (66.7)	0.2587
≥ 200 U/mL	14 (58.3)	10 (41.7)	
HE4 (pmol/L)			
< 140 pmol/L	5 (38.5)	8 (61.5)	0.296
≥ 140 pmol/L	12 (60)	8 (40)	
Ascites			
No	2 (28.6)	5 (71.4)	0.2245
Yes	15 (57.7)	11 (42.3)	

*TILs* Tumour-infiltrating lymphocytes, *OC* Ovarian cancer, *FIGO* The international federation of gynecology and obstetrics

Data was analysed by Chi-square or Fisher exact test. * *P* value in bold indicates statistically significant

### Proportion of CD4^+^CCR8 Tregs in infiltrating CD4^+^ Tregs in OC tissues

**Fig. 2:**
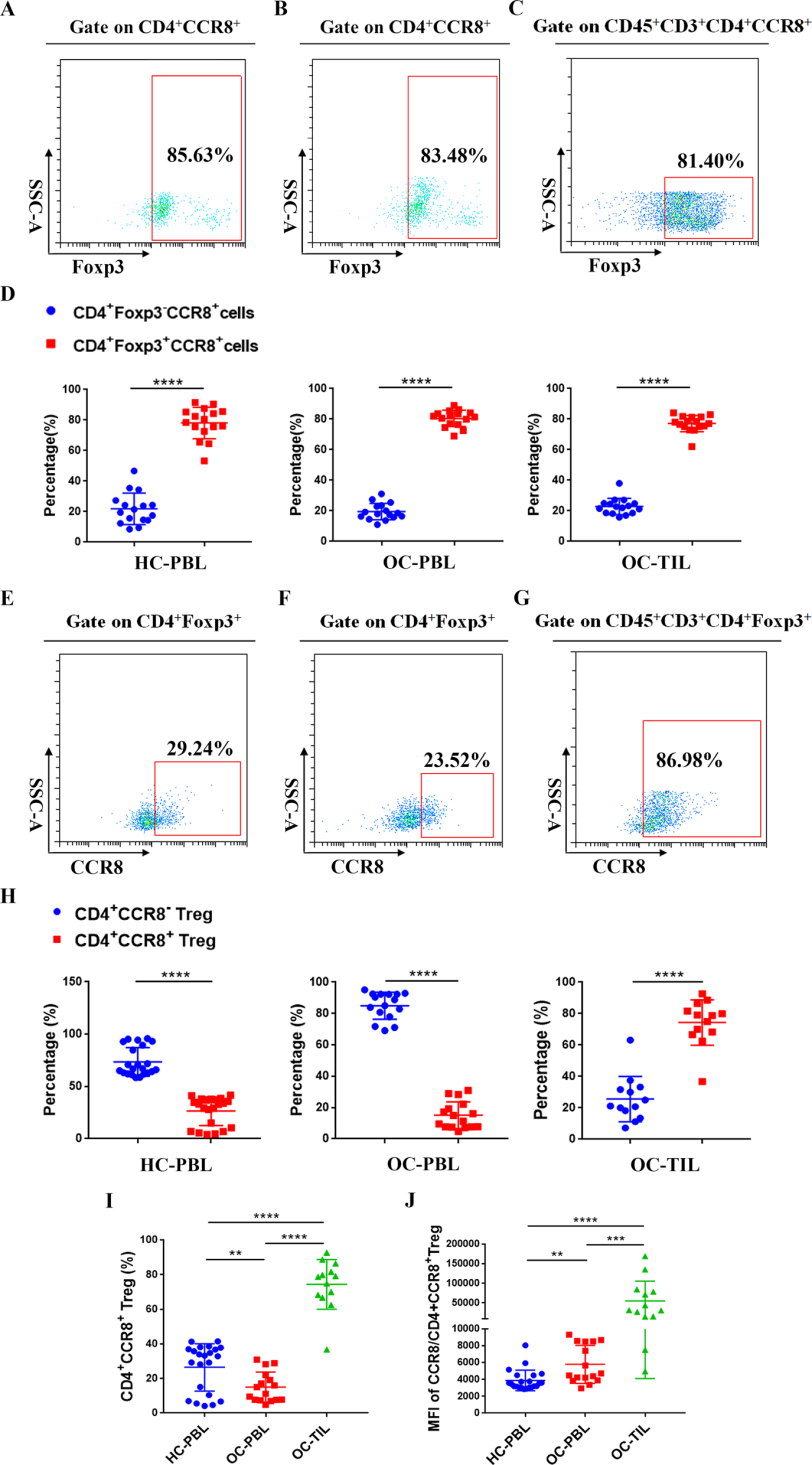
The proportion of CD4^+^Foxp3^−^CCR8^+^/CD4^+^Foxp3^+^CCR8^+^ cells, CD4^+^CCR8^−^/CD4^+^CCR8^+^ Tregs and the mean fluorescence intensity of CCR8 on HC-PBL, OC-PBL and OC-TIL groups **A** Representative flow cytometry analysis of CD4^+^Foxp3^−^CCR8^+^/CD4^+^Foxp3^+^CCR8^+^ cells in HC-PBL group (Foxp3 was used for gating CD4^+^CCR8^+^ cells). **B** Representative flow cytometry analysis of CD4^+^Foxp3^−^CCR8^+^/CD4^+^Foxp3^+^CCR8^+^ cells in OC-PBL group (Foxp3 was used for gating CD4^+^CCR8^+^ cells). **C** Representative flow cytometry analysis of CD4^+^Foxp3^−^CCR8^+^/CD4^+^Foxp3^+^CCR8^+^ cells in OC-TIL group (Foxp3 was used for gating CD45^+^CD3^+^CD4^+^CCR8^+^ cells). **D** Statistical chart of the percentage of CD4^+^Foxp3^−^CCR8^+^/CD4^+^Foxp3^+^CCR8^+^ cells in HC-PBL (n = 16), OC-PBL (n = 16) and OC-TIL (n = 16) groups. **E** Representative flow cytometry analysis of CD4^+^CCR8^−^/CD4^+^CCR8^+^ Tregs in HC-PBL group (CCR8 was used for gating CD4^+^Foxp3^+^ cells). **F** Representative flow cytometry analysis of CD4^+^CCR8^−^/CD4^+^CCR8^+^ Tregs in OC-PBL group (CCR8 was used for gating CD4^+^Foxp3^+^ cells). **G** Representative flow cytometry analysis of CD4^+^CCR8^−^/CD4^+^CCR8^+^ Tregs in OC-TIL group (CCR8 was used for gating CD45^+^CD3^+^CD4^+^Foxp3^+^ cells). **H** Statistical chart of the proportion of CD4^+^CCR8^−^/CD4^+^CCR8^+^ Tregs in HC-PBL (n = 22), OC-PBL (n = 16) and OC-TIL (n = 13) groups. **I** Statistical chart of the proportion of CD4^+^CCR8 ^+^ Tregs in HC-PBL (n = 22), OC-PBL (n = 16) and OC-TIL (n = 13) groups. **J** Statistical chart of the mean fluorescence intensity (MFI) of CCR8 on HC-PBL (n = 22), OC-PBL (n = 16) and OC-TIL (n = 13) groups. Data are presented as means ± SD and analysed with Student’s *t*-test. (**P < 0.01; ***P < 0.001; ****P < 0.0001)

To further clarify the distribution characteristics of CCR8, we used flow cytometry to determine the proportions of Foxp3^−^ and Foxp3^+^ cell subsets in CD4^+^CCR8^+^ T cells from 16 OC patients and 16 healthy individuals (Fig. 2A–C). Statistical analysis results showed that Foxp3^+^ cell subset was the main component of CD4^+^CCR8^+^ T cells in all three groups (Fig. 2D), suggesting that CCR8 is mainly expressed on Tregs. Therefore, in the following experiments, we mainly explored the expression characteristics of CCR8 on Tregs of different sample types. Flow cytometry was used to detect the proportion of CD4^+^CCR8^−^ Tregs/CD4^+^CCR8^+^ Tregs and the mean fluorescence intensity (MFI) of CCR8 expression in HC-PBL group (n = 22), OC-PBL group (n = 16) and OC-TIL group (n = 13), respectively (Fig. 2E–G). Statistical analysis showed that the proportion of CD4^+^CCR8^−^ Tregs in HC-PBL group and OC-PBL group were significantly higher than that of CD4^+^CCR8^+^ Tregs (*P* < 0.0001). However, the proportion of CD4^+^CCR8^+^ Tregs in OC-TIL group was significantly higher than that in CD4^+^CCR8^−^ Tregs (Fig. 2H) (*P* < 0.0001). Moreover, as shown in Fig. 2I, the proportion of CD4^+^CCR8^+^ Tregs in OC-TIL group was significantly higher than that in the other two groups (*P* < 0.0001). Subsequently, statistical analysis was performed on the MFI of CCR8 expression on CD4^+^CCR8^+^ Tregs in the three groups, as shown in Fig. 2J. The MFI of CCR8 in OC-TIL group was significantly higher than that in OC-PBL group and HC-PBL group (*P* < 0.001). These results suggest that CD4^+^CCR8^+^ Tregs are the main type of infiltrating CD4^+^ Tregs in OC tissues, and CD4^+^CCR8^−^ Tregs are the main component of CD4^+^ Tregs in peripheral blood. Compared with circulating CD4^+^ Tregs, the composition of infiltrating CD4^+^ Tregs in OC tissues was changed, and the proportion of infiltrating CD4^+^CCR8^+^ Tregs was increased and the expression intensity of CCR8 was enhanced.

### Immunosuppressive function and proliferative ability of infiltrating CD4^+^CCR8^+^ Tregs in OC tissues

**Fig. 3:**
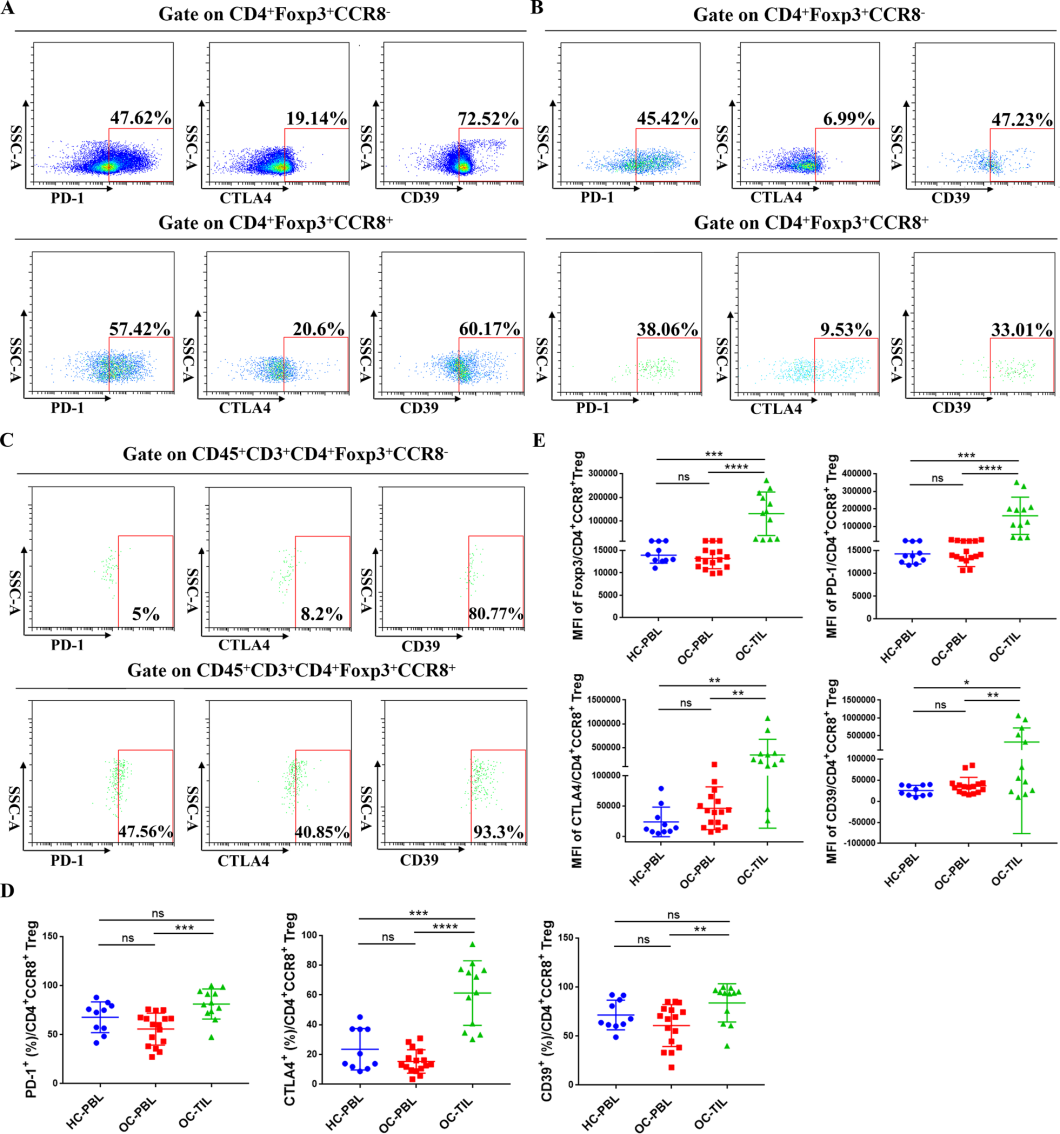
Phenotypic characteristics of CD4^+^CCR8^−^/CCR8^+^ Tregs in HC-PBL group, OC-PBL group and OC-TIL group **A** Representative flow cytometry analysis of inhibitory phenotype molecules of CD4^+^CCR8^−^/CCR8^+^ Tregs in HC-PBL group. **B** Representative flow cytometry analysis of inhibitory phenotype molecules of CD4^+^CCR8^−^/CCR8^+^ Tregs in OC-PBL group. **C** Representative flow cytometry analysis of inhibitory phenotype molecules of CD4^+^CCR8^−^/CCR8^+^ Tregs in OC-TIL group. **D** Statistical chart of the proportion of inhibitory phenotypic molecules in CD4^+^CCR8^+^ Tregs in HC-PBL (n = 10), OC-PBL (n = 16) and OC-TIL (n = 12) groups. **E** Statistical chart of the mean fluorescence intensity (MFI) of inhibitory phenotypic molecules in CD4^+^CCR8^+^ Tregs in HC-PBL (n = 10), OC-PBL (n = 16) and OC-TIL (n = 12) groups. Data are presented as means ± SD and analysed with Student’s *t*-test. (*P < 0.05; **P < 0.01; ***P < 0.001)

Typical inhibitory markers on the surface of Tregs include PD-1, CTLA-4, CD39 and so on, which can inhibit the activation of effector T cells by blocking stimulatory signals and producing inhibitory metabolites, and are related to the inhibitory function of Tregs. To further compare the phenotypic molecular characteristics of CD4^+^CCR8^+^ Tregs in peripheral circulation and that infiltrated in tumor tissues, flow cytometry was used to detect the proportions of PD-1^+^, CTLA4^+^, CD39^+^ cell subsets and the MFI values of Foxp3, PD-1, CTLA4 and CD39 on CD4^+^CCR8^−^ Tregs and CD4^+^CCR8^+^ Tregs from HC-PBL group (n = 10), OC-PBL group (n = 16) and OC-TIL group (n = 12) (Fig. 3A–C). As shown in Fig. 3D, the proportions of PD-1^+^ and CTLA4^+^ cell subsets of CD4^+^CCR8^+^ Tregs in OC-TIL group were significantly higher than that in OC-PBL group, while the proportion of CTLA4^+^ cell subsets of CD4^+^CCR8^+^ Tregs in OC-TIL group was significantly higher than that in HC-PBL group, and all the differences were statistically significant (*P* < 0.01). As shown in Fig. 3E, the MFI values of Foxp3, PD-1 and CTLA4 on CD4^+^CCR8^+^ Tregs from OC-TIL group was significantly higher than that from HC-PBL group and OC-PBL group, respectively. All the differences were statistically significant (*P* < 0.05).

**Fig. 4:**
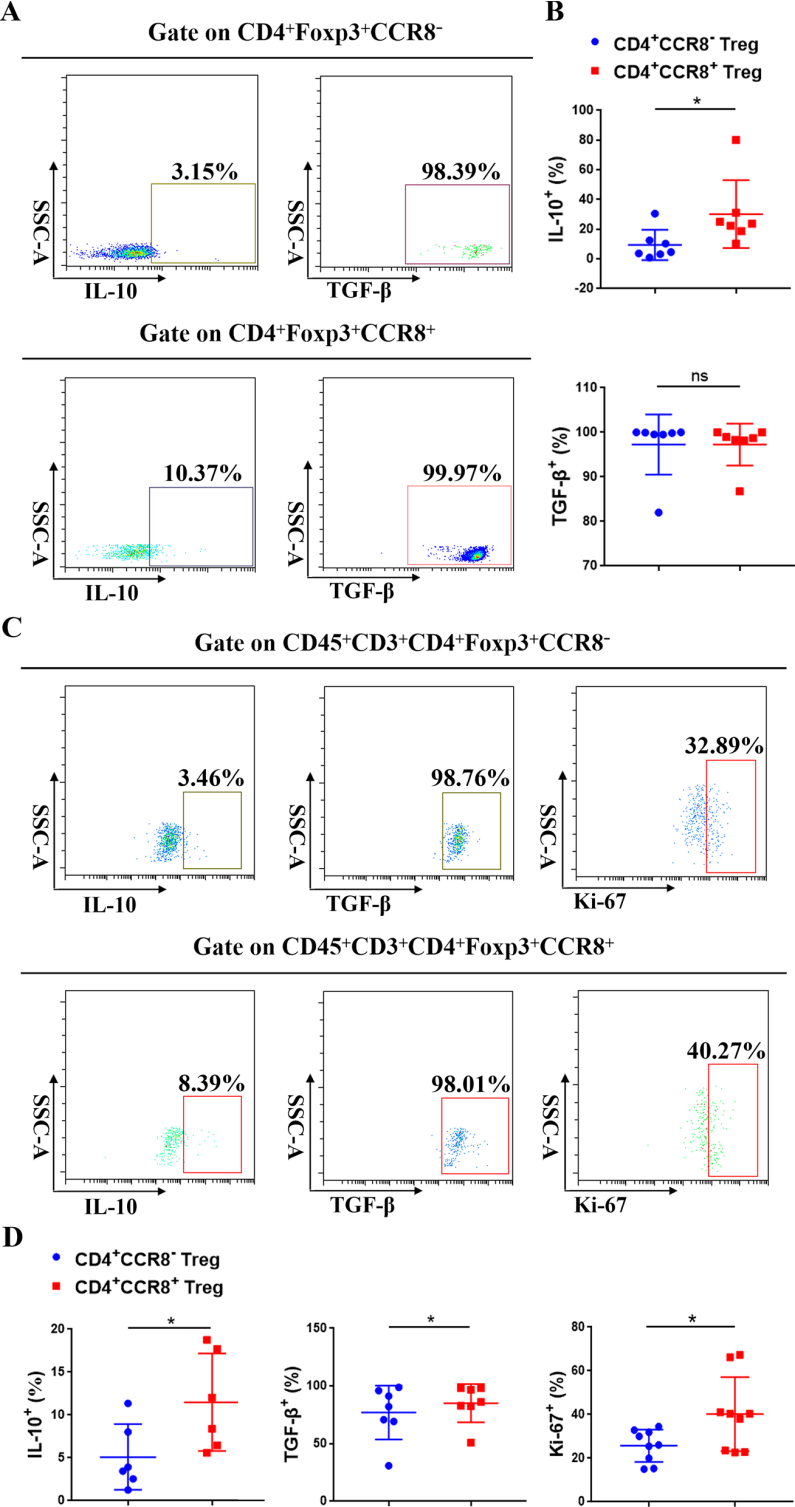
The expression levels of IL-10 and TGF-β in CD4^+^CCR8^−^/CCR8^+^ Tregs and the comparison of their proliferation ability** A** Representative flow cytometry analysis of IL-10 and TGF-β in CD4^+^CCR8^−^/CCR8^+^ Tregs of OC-PBL group. **B** Statistical chart of the positive proportion IL-10 (n = 7) and TGF-β (n = 7) in CD4^+^CCR8^−^/CCR8^+^ Tregs of OC-PBL group. **C** Representative flow cytometry analysis of IL-10, TGF-β and Ki-67 in CD4^+^CCR8^−^/CCR8^+^ Tregs of OC-TIL group. **D** Statistical chart of the positive proportion of IL-10 (n = 6), TGF-β (n = 7) and Ki-67 (n = 9) in CD4^+^CCR8^−^/CCR8^+^ Tregs of OC-TIL group. Data are presented as means ± SD and analysed with the with Student’s *t*-test. (*P < 0.05; **P < 0.01)

Previous studies have shown that Tregs could directly destroy T cell function by secreting a variety of inhibitory cytokines, which is an important reason for tumor cells to evade immune surveillance and ultimately lead to tumor formation, invasion and metastasis. In this study, flow cytometry was used to detect and analysis the expression of IL-10 and TGF-β of CD4^+^CCR8^−^ Tregs and CD4^+^CCR8^+^ Tregs in OC patients, as well as the comparison of the cell proliferative ability between them (Fig. 4A, C). Statistical analysis showed that the expression of IL-10 in CD4^+^CCR8^+^ Tregs was significantly higher than that of CD4^+^CCR8^−^ Tregs in OC-PBL group (n = 7) and OC-TIL group (n = 6), while the expression of TGF-β in CD4^+^CCR8^+^ Tregs was significantly higher than that of CD4^+^CCR8^−^ Tregs in OC-TIL group (n = 7), and infiltrating CD4^+^CCR8^+^ Tregs in OC tissues had stronger proliferative ability than CD4^+^CCR8^−^ Tregs (n = 9) (Fig. 4B, D).

### The role of CCR8-CCL1/CCL18 axis in the migration of CD4^+^CCR8^+^ Tregs into OC tissues

**Fig. 5:**
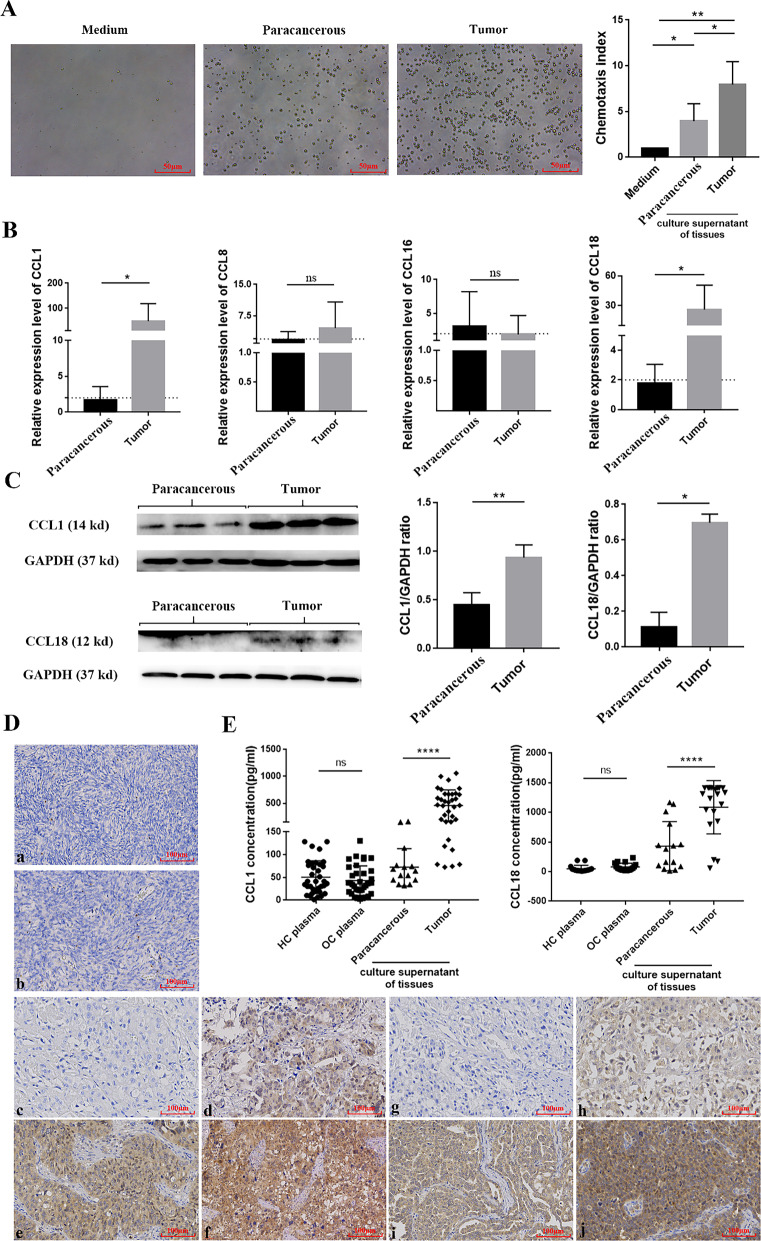
Chemotactic experiment of CD4^+^CCR8^+^ Tregs and expression levels of CCR8 ligands in ovarian tumor and paracancerous tissues **A** Chemotaxis assay of CD4^+^CCR8^+^ Tregs in medium group, paracancerous tissue culture supernatant group and OC tissue culture supernatant group (original magnification 200 ×). **B** Gene expression levels of CCL1, CCL8, CCL16 and CCL18 in ovarian tumor (n = 32) and paracancerous tissues (n = 32). **C** The protein expression levels of CCL1 and CCL18 in ovarian tumor and paracancerous tissues. **D** Expression levels of CCL1 and CCL18 in ovarian tumor and paracancerous tissues (original magnification 400 ×). **a** Expression of CCL1 in paracancerous tissues; **b** Expression of CCL18 in paracancerous tissues; **c–f** The expression of CCL1 in OC tissue was 0 (−), 4 ( +), 8 (+ +), 12 (+ + +). **g–h** The expression of CCL18 in OC tissue was 0 (−), 4 ( +), 8 (+ +), 12 (+ + +). **E** The contents of CCL1 in the plasma of healthy individuals (n = 35), the plasma of OC patients (n = 36), the culture supernatants of ovarian tumor (n = 15) and paracancerous tissues (n = 35); the contents of CCL18 in the plasma of healthy individuals (n = 17), the plasma of OC patients (n = 18), the culture supernatants of ovarian tumor (n = 15) and paracancerous tissues (n = 20). Each experiment was performed triplicated. Data are presented as means ± SD and analysed with Student’s *t*-test. (*P < 0.05; **P < 0.01; ****P < 0.0001)

In order to investigate the reason for the increased infiltration of CD4^+^CCR8^+^ Tregs in OC tissues, CD4^+^CCR8^+^ Tregs from peripheral blood were co-cultured with the supernatants of OC tissues and paracancerous tissues, respectively, using 1640 Medium as a control. Chemotaxis index was calculated for each group, and it indicated that chemotaxis in group co-cultured with OC tissue cultured supernatant was significantly stronger towards CD4^+^CCR8^+^ Tregs than that in group co-cultured with paracancerous tissue cultured supernatant (Fig. 5A), suggesting that there may be some components in the tumor tissue microenvironment that can drive CD4^+^CCR8^+^ Tregs to the tumor tissues. At present, there are four known ligands for human CCR8: CCL1, CCL8, CCL16, and CCL18. We first detected the expression of the above four ligands in OC tissues (n = 32) and paracancerous tissues (n = 32) by RT-PCR, and the results were shown in Fig. 5B. It was indicated that the expression of CCL1 and CCL18 in OC tissues were significantly higher than that in paracancerous tissues, while there was no significant difference in the expression of CCL8 and CCL16 between them. Moreover, following assays of western blot, immunohistochemical staining and ELISA all confirmed the above conclusion in multiple aspects (Fig. 5C–E).

**Fig. 6:**
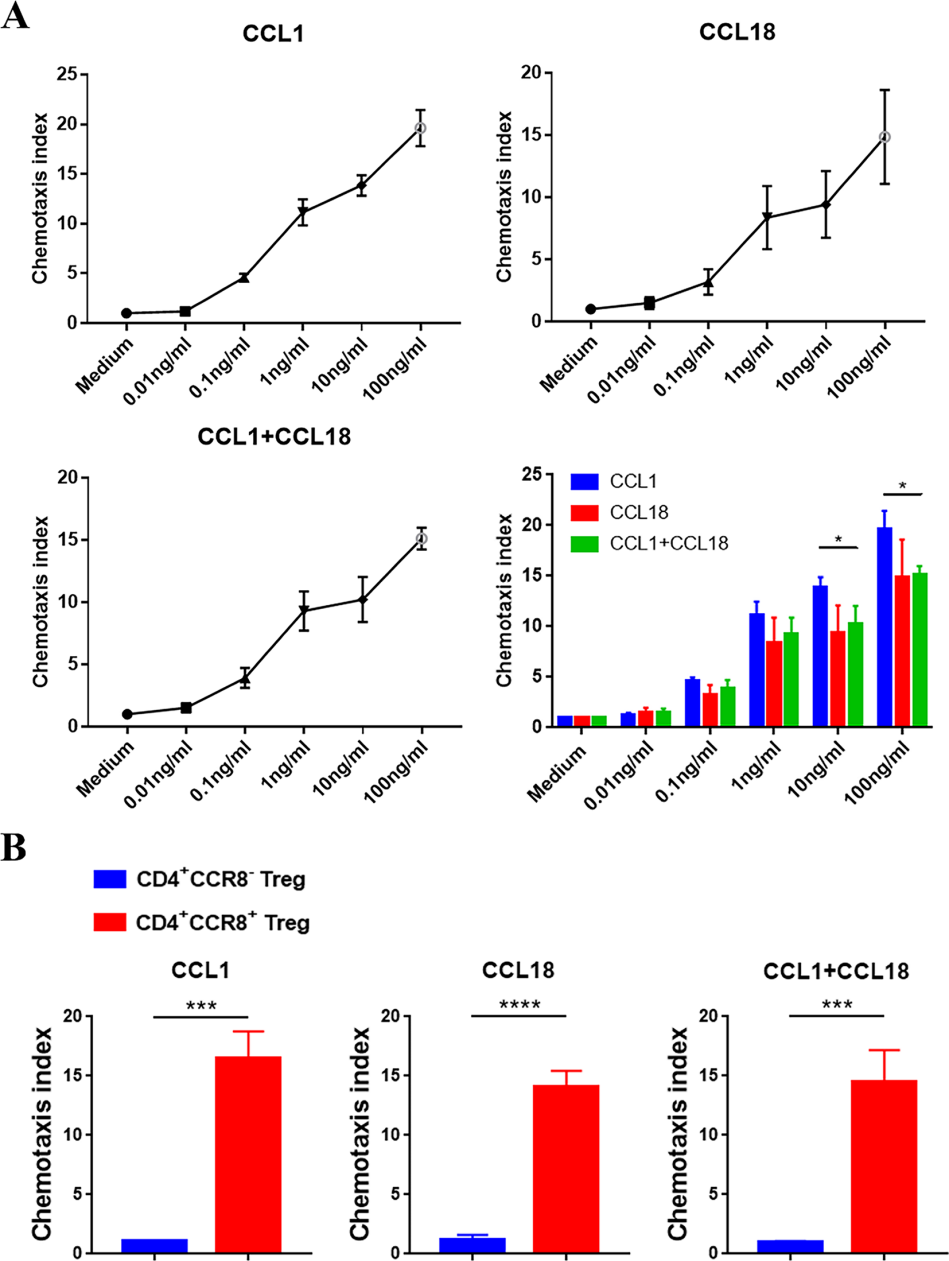
In vitro chemotaxis experiment of CD4^+^CCR8^−^/CCR8^+^ Tregs** A** Comparative analysis of the difference of chemotactic ability of CD4^+^CCR8^+^ Tregs in the medium containing gradient concentration CCL1/CCL18. **B** Comparative analysis of the difference of response chemotaxis between CD4^+^CCR8^−^ Tregs and CD4^+^CCR8^+^ Tregs. Each experiment was performed triplicated. Data are presented as means ± SD and analysed with the non-parametric Mann–Whitney U test. (*P < 0.05)

In order to investigate whether the high concentration of CCL1 and CCL18 in OC tissues acts as a participation factor to independently or cooperatively mediate the chemotaxis of CD4^+^CCR8^+^ Tregs, CD4^+^CCR8^+^ Tregs were co-cultured with culture-mediums containing chemokines CCL1 and/or CCL18 with gradient concentrations (0.01 ng/mL, 0.1 ng/mL, 1 ng/mL, 10 ng/mL and 100 ng/mL), and then the chemotactic index of each group was calculated. As shown in Fig. 6A, chemotaxis towards CD4^+^CCR8^+^ Tregs could be observed when the mediums contained chemokines CCL1 and CCL18 with gradient concentration alone or concurrently, and the chemotactic index was elevated with the increase of chemokine concentration within the set concentration range. These results suggested that the difference in the concentration of CCL1 and/or CCL18 in OC tissues, paracancerous tissues and peripheral blood may lead to the recruitment of CD4^+^CCR8^+^ Tregs from paracancerous tissues or peripheral blood into OC tissues, thus leading to increased infiltration of CD4^+^CCR8^+^ Tregs in OC tissues. Subsequently, we co-cultured CD4^+^CCR8^−^ Tregs and CD4^+^CCR8^+^ Tregs with mediums containing 100 ng/mL CCL1 and/or 100 ng/mL CCL18, respectively, and chemotactic index of each group was calculated. Figure 6B showed that chemotactic index of CD4^+^CCR8^+^ Tregs were significantly higher than that of CD4^+^CCR8^−^ Tregs in all three groups, while CD4^+^CCR8^−^ Tregs showed rare chemotaxis, suggesting CCR8 as a key molecule on CD4^+^CCR8^+^ Tregs in response to chemotactic signals.

## Discussion

T lymphocytes infiltration is a common feature in many solid tumors and TILs can be classified according to their function and location in tumor tissues, which play a central role in tumor immune response and are closely related to the treatment and prognosis of patients with multiple tumor types [23–36]. TILs are mainly derived from peripheral blood, and once they penetrate into tumor sites, their functions change greatly, including their differentiation stage, surface markers, and secretion types [37, 38]. We hypothesize that the molecular basis of these functional differences in TILs is due to changes in their expression profiles. Therefore, we analyzed mRNA expression profiles of CD4^+^ T cells isolated from peripheral blood of healthy individuals, peripheral blood of OC patients and tumor tissues of OC patients, and then found that differential mRNAs in the three groups were significantly enriched in the chemokine-chemokine receptor signaling pathway, suggesting that this pathway may be widely involved in the regulation of biological functions of CD4^+^ T cells in different locations.

Different immune cell subsets can reshape the immunophenotype of the tumor immune microenvironment through cell migration mediated by chemokine-chemokine receptor signals, so as to regulate tumor immunity and tumor formation [39–42]. Previous studies have shown that chemokines CXCL9 and CXCL10 can recruit CD8^+^ T cells, Th1 cells and NK cells by recognizing CXCR3 signals, thus triggering anti-tumor response [43]. Various types of chemokine receptors can be expressed on the surface of CD4^+^ T cells. In this study, chemokine receptor-related differential genes were selected for further in vitro verification and CCR8 was identified as the target gene. Further analysis showed that the CCR8 expression on infiltrating CD4^+^ T cells in OC tissues was significantly higher than that of peripheral blood of healthy individuals and OC patients which was significantly correlated with advanced stage and poor differentiation.

Tregs are key drivers of immunosuppression in solid tumors. It has been reported that CCR8 can be used as a specific marker which could be selectively upregulated on infiltrating Tregs in a variety of cancers, including lung, breast, colorectal cancer and so on [44]. Increased CCR8^+^ Tregs infiltration is associated with more advanced stages of the disease and decreased overall survival of patients [45–47]. These findings reflect the therapeutic potential of CCR8 as a target for Tregs resident in tumors. In this study, we found that CD4^+^CCR8^+^ Tregs are the main type of CD4^+^ Tregs in OC tissues, and the expression of chemokine receptor (CCR8), inhibitory phenotypes (PD-1, CTLA4, CD39), inhibitory cytokines (IL-10, TGF-β) and proliferation ability of infiltrating CD4^+^CCR8^+^ Tregs in OC tissues were all enhanced, indicating stronger immunosuppressive function. The phenotype of the same type of cells can be changed depending on the TME, indicating the remodeling effect of TME on infiltrating cells.

In addition, we found that ovarian tumor tissue culture supernatant had a more significant chemotactic effect on CD4^+^CCR8^+^ Tregs, suggesting that some components of the culture supernatant were involved in recruitment. According to literature, chemokines CCL1, CCL8, CCL16 and CCL18 are ligands corresponding to CCR8 [40]. Based on this, we found that CCL1 and CCL18 are highly expressed in OC tissues. Further chemotactic experiments in vitro also confirmed that CCL1 and CCL18 had a positive effect on the recruitment of CD4^+^CCR8^+^ Tregs. Therefore, we believe that the high expression of CCL1 and CCL18 in ovarian cancer TME is an important factor in the increase of CD4^+^CCR8^+^ Tregs infiltration into tumor tissues.

Overall, high levels of CCL1 and CCL18 in OC tissues can recruit CD4^+^CCR8^+^ Tregs into tumor tissues by recognizing CCR8 on cells and remodeling their inhibitory phenotypes, making them have stronger immunosuppressive phenotypes and proliferative abilities. However, the specific mechanisms underlying changes in phenotypes and immunosuppressive functions of CD4^+^CCR8^+^ Tregs recruited into tumor tissues are still unclear and need to be further studied. The immunosuppressive cell population in TME prevents the immune system from effectively destroying tumor cells. Targeting chemokine receptors on the suppressive cells in tumor tissues to selectively reduce the number, frequency, or function can enhance the anti-tumor immune response and contribute to the treatment of ovarian cancer, which may be the rock of combined immunotherapy strategies in future.

## Conclusion

There were significant differences in gene expression profiles between peripheral circulating CD4^+^ T cells and infiltrating CD4^+^ T cells in OC tissues. In the TME of ovarian cancer, CCR8 was the dominant chemokine receptor expressed on CD4^+^ TILs which was closely related to the pathological process of ovarian cancer. The inhibitory phenotypes and proliferative abilities of infiltrating CD4^+^CCR8^+^ Tregs in OC tissues were significantly increased, and more inhibitory cytokines would be secreted. Chemotactic assays in vitro suggested that high level of CCL1 and CCL18 in the TME of ovarian cancer contributed to the increased infiltration of CD4^+^CCR8^+^ Tregs into tumor tissues. CCR8^+^ tumor-resident Tregs could be used as a reasonable target for cancer immunotherapy, and provide a new idea for selectively targeting intratumoral dysfunction or cell depletion of Tregs.

### Supplementary Information

**Additional file 1: Figure S1.** Transcriptome analysis between CD4^+^ PBLs and CD4^+^ TILs **A** Scatter plot analysis of differentially expressed mRNA in OC-PBL vs HC-PBL group, OC-TIL vs HC-PBL group and OC-TIL vs OC-PBL group. The horizontal coordinates indicate the mean expression values of the samples in group A and the vertical coordinates indicate the mean expression values of the samples in group B. **B** Sample correlation analysis in HC-PBL, OC-PBL and OC-TIL groups. The sample correlation matrix is the pearson correlation coefficient matrix between samples, and the value in the matrix is a decimal of 0-1, indicating the pearson correlation coefficient of two corresponding samples. The sample correlation graph directly reflects the similarity between samples. The value of each cell in the lower left is the correlation coefficient of the corresponding two samples, and the color and area of the circle in the upper right represent the correlation degree of the corresponding samples. The larger the circle area, the higher the correlation between the two samples. **C** Enrichment analysis of GO functional pathways differentially expressed mRNA in OC-PBL vs HC-PBL group, OC-TIL vs HC-PBL group and OC-TIL vs OC-PBL group. **D** Enrichment analysis of KEGG signaling pathway for differentially expressed mRNA in OC-PBL vs HC-PBL group, OC-TIL vs HC-PBL group and OC-TIL vs OC-PBL group. (HC-PBL group: CD4+ PBLs from healthy individuals; OC-PBL group: CD4+ PBLs from OC patients; OC-TIL group: CD4+ TILs from OC patients.)

**Additional file 2.** **Figure S2.** Graphical Abstract.

**Additional file 3.** **Figure S3.** Flow chart of research strategy.

**Additional file 4: Table S1.** List of the chemokine receptor-related differential genes.

**Additional file 5: Table S2.** Correlations between the expression of CCR8 on CD4^+^ PBLs and the clinicopathologic characteristics of OC patients.

## Data Availability

All the data generated and utilized in this study are included in this published article.

## Abbreviations

OC: Ovarian cancer
PBMCs: Peripheral blood mononuclear cells
TILs: Tumour-infiltrating lymphocytes
PBLs: Peripheral blood lymphocytes
FC: Fold change
