# Supplementary figures and images for: CD4+CCR8+ Tregs in ovarian cancer: a potential effector Tregs for immune regulation
Source: J Transl Med. 2023 Nov 10;21:803. doi: 10.1186/s12967-023-04686-3 (PMC10638792; doi:10.1186/s12967-023-04686-3)

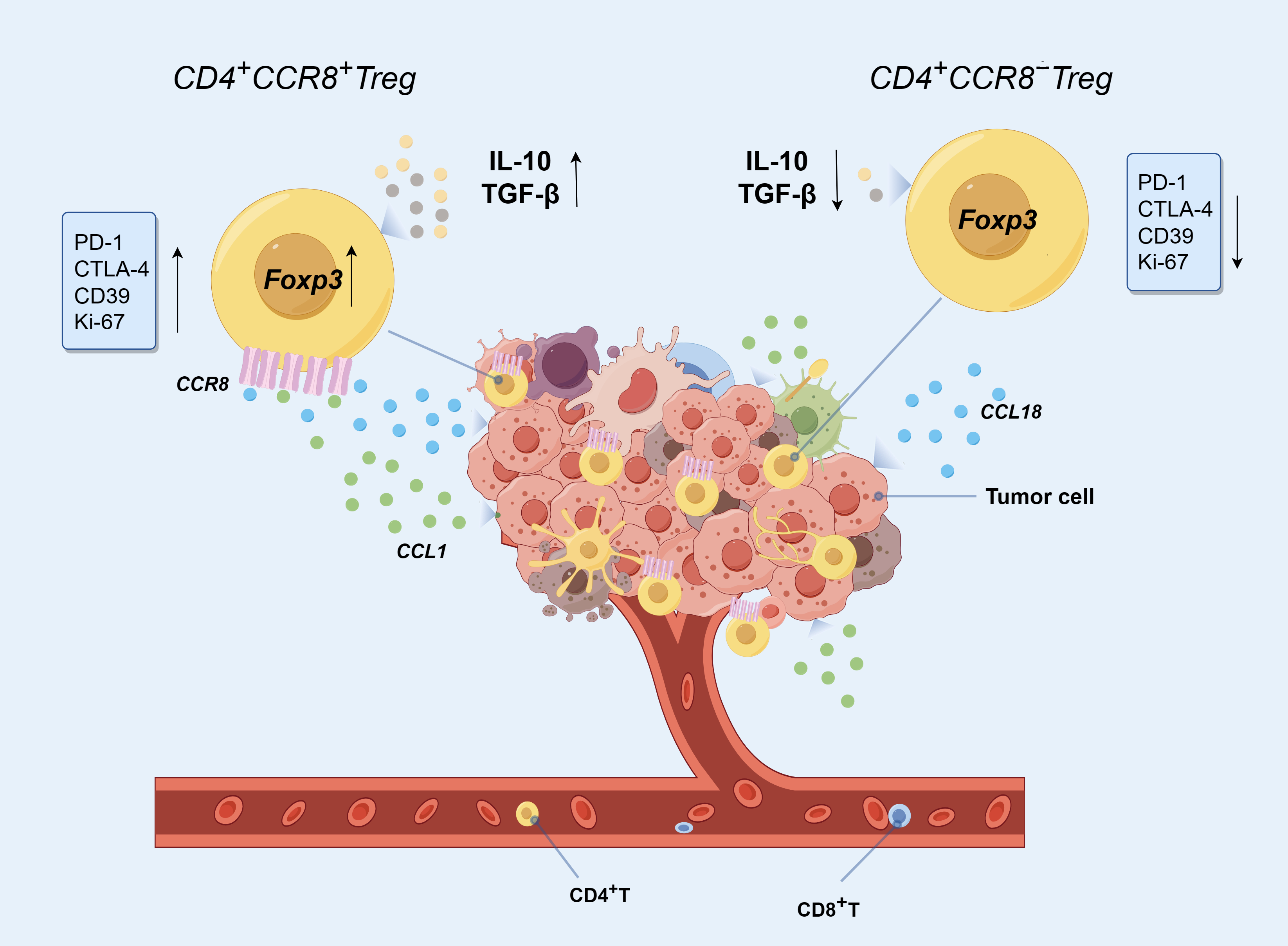

Supplement: Supplementary file 2 — Additional file 2. Figure S2. Graphical Abstract. [file 12967_2023_4686_MOESM2_ESM.tif]

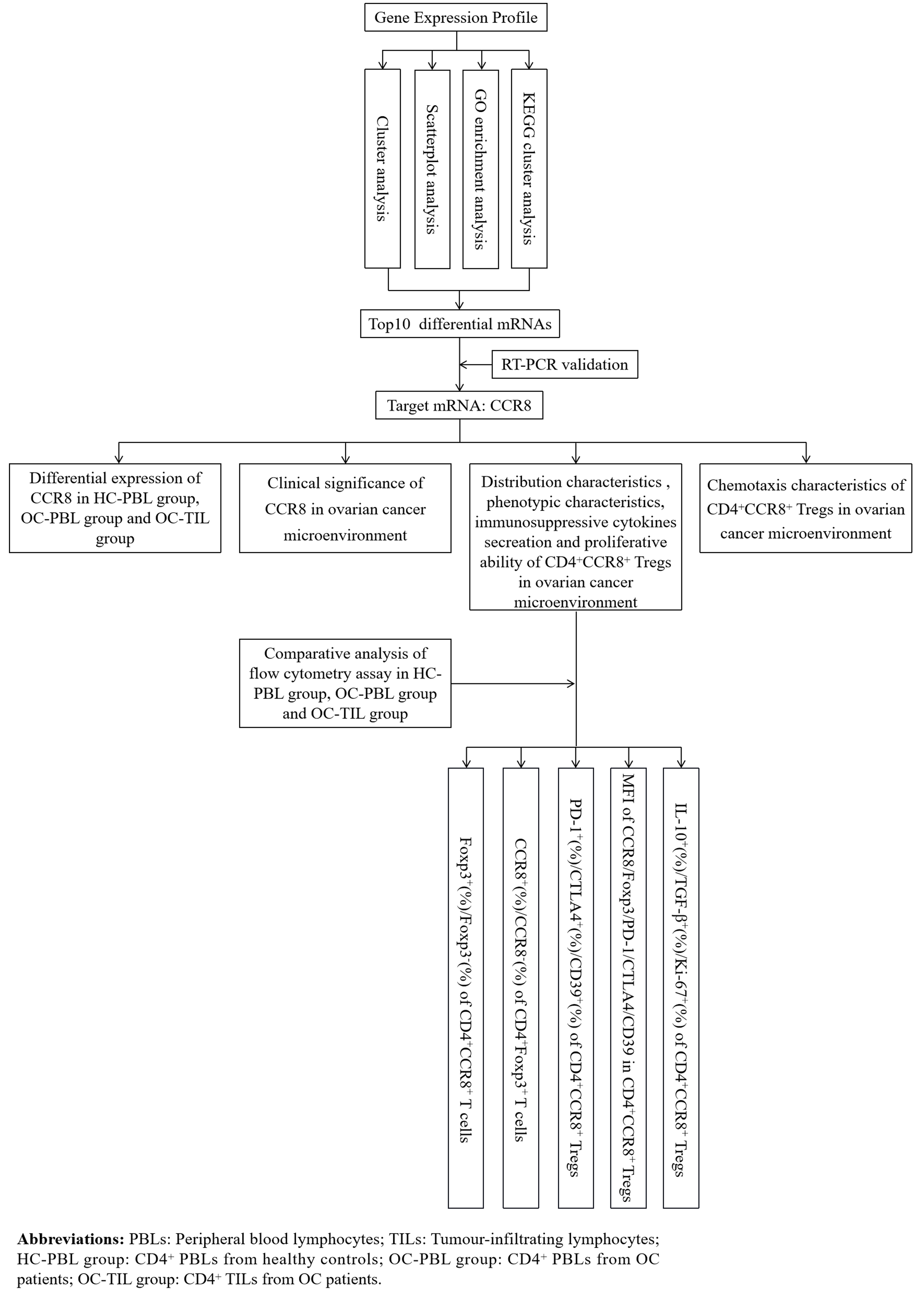

Supplement: Supplementary file 3 — Additional file 3. Figure S3. Flow chart of research strategy. [file 12967_2023_4686_MOESM3_ESM.tif]
