# Supplementary material for: CD4+CCR8+ Tregs in ovarian cancer: a potential effector Tregs for immune regulation
Source: J Transl Med. 2023 Nov 10;21:803. doi: 10.1186/s12967-023-04686-3 (PMC10638792; doi:10.1186/s12967-023-04686-3)
Supplement: Supplementary file 4 — Additional file 4: Table S1. List of the chemokine receptor-related differential genes. [file 12967_2023_4686_MOESM4_ESM.docx]

**Table S1. List of the chemokine receptor-related differential genes**

| **Group** | **ProbeName** | ***P*** | **FC (abs)** | **Regulation** | **GeneSymbol** |
| --- | --- | --- | --- | --- | --- |
| **OC-P vs HC** | A_24_P148717 | 0.000190375 | 10.57248906 | up | CCR1 |
|  | A_23_P114299 | 0.006096553 | 2.697740992 | down | CXCR3 |
|  | A_24_P252945 | 0.001310418 | 2.229856289 | down | CXCR5 |
| **OC-T vs HC** | A_24_P148717 | 0.000369305 | 10.729547 | up | CCR1 |
|  | A_23_P324885 | 0.022503708 | 4.094175281 | up | CCR2 |
|  | A_23_P212354 | 0.045892199 | 2.925601842 | up | CCR2 |
|  | A_23_P412321 | 0.000064711 | 8.482544731 | up | CCR5 |
|  | A_33_P3313929 | 0.005158375 | 2.202507009 | down | CCR6 |
|  | A_23_P343398 | 0.000000017 | 14.02245144 | down | CCR7 |
|  | A_23_P69012 | 0.000000314 | 73.44251238 | up | CCR8 |
|  | A_33_P3395595 | 0.000076829 | 12.58160865 | down | CCR9 |
|  | A_33_P3221303 | 0.000019810 | 4.150295821 | down | CCR10 |
|  | A_23_P102000 | 0.001012806 | 2.517498549 | down | CXCR4 |
|  | A_24_P252945 | 0.000012161 | 9.273763392 | down | CXCR5 |
|  | A_23_P109913 | 0.000062227 | 8.969472312 | up | CXCR6 |
| **OC-T vs OC-P** | A_23_P250302 | 0.008327046 | 2.105678815 | up | CCR3 |
|  | A_23_P412321 | 0.000143162 | 10.83741418 | up | CCR5 |
|  | A_23_P343398 | 0.000000029 | 10.82855071 | down | CCR7 |
| **OC-T vs OC-P** | A_23_P69012 | 0.000000002 | 110.9302597 | up | CCR8 |
|  | A_33_P3395595 | 0.000003891 | 12.31083209 | down | CCR9 |
|  | A_33_P3221303 | 0.000000609 | 4.462917178 | down | CCR10 |
|  | A_23_P114299 | 0.000972678 | 3.648901889 | up | CXCR3 |
|  | A_24_P252945 | 0.000120511 | 4.158906311 | down | CXCR5 |
|  | A_23_P109913 | 0.000013817 | 8.244934698 | up | CXCR6 |
