# Supplementary material for: CD4+CCR8+ Tregs in ovarian cancer: a potential effector Tregs for immune regulation
Source: J Transl Med. 2023 Nov 10;21:803. doi: 10.1186/s12967-023-04686-3 (PMC10638792; doi:10.1186/s12967-023-04686-3)
Supplement: Supplementary file 5 — Additional file 5: Table S2. Correlations between the expression of CCR8 on CD4+ PBLs and the clinicopathologic characteristics of OC patients. [file 12967_2023_4686_MOESM5_ESM.docx]

**Table S2. Correlations between the expression of CCR8 on CD4^+^ PBLs and the clinicopathologic characteristics of OC patients**

| **Clinical Variables** | **CCR8^low^ (%)** | **CCR8^high^ (%)** | ***P* value** |
| --- | --- | --- | --- |
| **Sample size** | 19 (48.7) | 20 (51.3) |  |
| **Age (year)** |  |  |  |
| <50 years | 7 (38.9) | 11 (61.1) | 0.3406 |
| ≥50 years | 12 (57.1) | 9 (42.9) |  |
| **Tumor size (cm)** |  |  |  |
| <5 cm | 4 (80) | 1 (20) | 0.1818 |
| ≥5 cm | 15 (44.1) | 19 (55.9) |  |
| - **Histologic type** |  |  |  |
| Serous carcinoma | 12 (44.4) | 15 (55.6) | 0.328 |
| Endometrioid cancer | 4 (80) | 1 (20) |  |
| Clear cell carcinom | 3 (42.9) | 4 (57.1) |  |
| **FIGO stage** |  |  |  |
| I-II | 9 (56.3) | 7 (43.8) | 0.5231 |
| III-IV | 10 (43.5) | 13 (56.5) |  |
| **Differentiation** |  |  |  |
| Well to moderate | 10 (58.8) | 7 (41.2) | 0.3406 |
| Poor | 9 (40.9) | 13 (59.1) |  |
| **Lymphatic metastasis** |  |  |  |
| No | 12 (57.1) | 9 (42.9) | 0.3406 |
| Yes | 7 (38.9) | 11 (61.1) |  |
| **Distant metastasis** |  |  |  |
| No | 8 (50) | 8 (50) | >0.9999 |
| Yes | 11 (47.8) | 12 (52.2) |  |
| **CA125 (U/mL)** |  |  |  |
| < 200 U/mL | 8 (53.3) | 7 (46.7) | 0.7475 |
| ≥ 200 U/mL | 11 (45.8) | 13 (54.2) |  |
| **HE4 (pmol/L)** |  |  |  |
| <140 pmol/L | 9 (52.9) | 8 (47.1) | 0.7512 |
| ≥140 pmol/L | 10 (45.5) | 12 (54.5) |  |
| **Ascites** |  |  |  |
| No | 4 (57.1) | 3 (42.9) | 0.6498 |
| Yes | 15 (46.9) | 17 (53.1) |  |

PBLs: Peripheral blood lymphocytes; OC: Ovarian cancer; FIGO: The International Federation of Gynecology and Obstetrics.

Data was analysed by Chi-square or Fisher exact test. * *P* value in bold indicates statistically significant.
